# Efficacy analysis of splint combined with platelet-rich plasma in the treatment of temporomandibular joint osteoarthritis

**DOI:** 10.3389/fphar.2022.996668

**Published:** 2022-11-14

**Authors:** Chuan-Bin Wu, Ning-Ning Sun, Dan Zhang, Qiang Wang, Qing Zhou

**Affiliations:** ^1^ Liaoning Provincial Key Laboratory of Oral Diseases, Department of Oral and Maxillofacial Surgery, School and Hospital of Stomatology, China Medical University, Shenyang, China; ^2^ Liaoning Provincial Key Laboratory of Oral Diseases, Department of Orthodontics, School and Hospital of Stomatology, China Medical University, Shenyang, China; ^3^ Liaoning Provincial Key Laboratory of Oral Diseases, School and Hospital of Stomatology, China Medical University, Shenyang, China

**Keywords:** PRP, temporomandibular joint, osteoarthritis, VAS, splint, mouth opening

## Abstract

**Objective:** To evaluate the efficacy of splints combined with PRP for the treatment of temporomandibular joint osteoarthritis.

**Methods:** Thirty-one patients with temporomandibular joint osteoarthritis who were treated with splints combined with platelet-rich plasma (PRP) from January 2021 to June 2021 at the Department of Oral and Maxillofacial Surgery, School of Stomatology, China Medical University (Shenyang, China) were retrospectively reviewed. The VAS scores of all the patients were recorded before and 6 months after treatment, and the maximum comfortable mouth opening was recorded. All data were analyzed by the paired *t*-test using SPSS software, and a *p*-value < 0.05 indicated statistically significant differences.

**Results:** Splint + PRP treatment was successful in 31 patients. The mean pretreatment VAS score was 6.1, and the mean VAS score 6 months posttreatment was 4.1. The posttreatment VAS score was significantly lower than the preoperative VAS score (*p* < 0.05). The mean pretreatment maximum comfortable mouth opening (MCMO) was 27.6 mm, and the mean MCMO 6 months posttreatment was 34.8 mm. The MCMO was significantly increased (*p* < 0.05).

**Conclusion:** Splint + PRP is an effective treatment for temporomandibular joint osteoarthritis.

## 1 Introduction

Temporomandibular joint osteoarthritis (TMJ-OA) is a common disease in the oral and maxillofacial regions and is the most serious type of temporomandibular disorder (TMD) (Lee et al., 2020). The incidence of TMD is high, impacting approximately 5%–12% of the population (Shoukri et al., 2019). Among TMDs, TMJ-OA accounts for 18%–85% of all cases (Figueiredo Valesan et al., 2021). TMJ-OA is a chronic and progressive disease that causes degeneration of the temporomandibular joint cartilage (Wang et al., 2015; Lee et al., 2020), thus pathologically characterized by degeneration, destruction, loss of articular cartilage, subchondral osteosclerosis, osteophyte formation, subosseous microcapsule formation, and accompanying varying degrees of synovial inflammation. The clinical symptoms of TMJ-OA include joint pain and joint clicking, which can eventually cause joint structure destruction, thus seriously affecting the quality of life of patients (Al-Ani, 2021; Sha-Sha et al., 2022).

Young patients who suffer from TMJ-OA are more likely to choose conservative treatment than surgical treatment (Kang, 2021). Postsurgical scars affect physical appearance. The facial nerve and parotid glands may also be damaged in surgery (Hoffman and Puig, 2015). Therefore, new and effective conservative treatment options are urgently needed.

Platelet-rich plasma (PRP) is extracted in high concentrations from the patient’s blood by centrifugation, and its concentration is 3–4 times that of platelets in human plasma. PRP has rich chondrogenic growth factors and can repair cartilage. The advantage of its application is that it is easy to obtain and prepare, and there are almost no potential safety hazards (Sha-Sha et al., 2022; I-Kung Wu and Borg-Stein, 2016; Everts et al., 2020).

A splint is commonly used in conservative treatment, has a relatively simple application, does not cause harm to the teeth or jaws, is noninvasive to the human body and can be worn by itself. The application process is reversible and easy to perform. A splint can be placed in the upper or lower jaw. After the splint is inserted, the position of the condylar process in relation to the glenoid fossa changes, which stabilizes the structure of the temporomandibular joint and increases the vertical distance between the maxilla and mandible, thus reducing the contraction burden of the masticatory muscles and relieving the hyperfunction of the muscles, which in turn causes the joints and muscles to relax, thus improving mouth opening capacity and relieving pain. The neuromuscular reflex control effect produced after wearing the splint can also reduce the internal stress of masticatory muscles and the temporomandibular joint, thus improving their functional status (Zhang et al., 2016; Crout, 2017).

Most patients with TMJ-OA have poor oral habits, such as chewing-side preference and bruxism, which transfer abnormal stress to the joint area (Su et al., 2018). Given such conditions, we researched the significance of stress-related proteins in TMD. Therefore, the removal of abnormal stress loading is important to TMJ-OA treatment. Splints are excellent tools used for the prevention of abnormal stress. Piezo1, a mechanically sensitive iron channel protein, is the focus of our research (Coste et al., 2010; Coste et al., 2012; Wu et al., 2022). We found that unbalanced stress may activate Piezo1, thus leading to TMJ-OA. On the other hand, the inflammatory environment may activate Piezo1, thus aggravating TMJ-OA (Velasco-Estevez et al., 2020; Lee et al., 2021). Given these results, we inferred that the inflammatory environment may lead to the development of TMJ-OA. Given such conditions, eliminating inflammation is another vital treatment strategy. PRP contains leukocyte components that exert an anti-inflammatory effect.

We led the implementation of splinting combined with PRP, a treatment modality, for the treatment of temporomandibular joint osteoarthritis. The aim of the study was to assess the therapeutic efficacy of Splint + PRP treatment in patients with TMJ-OA. The efficacy analysis is summarized as follows:

## 2 Materials and methods

### 2.1 Study subjects and groups

Thirty-one patients with TMJ-OA who came to the Affiliated Stomatological Hospital of China Medical University between January 2021 and June 2021 were selected and treated with Splint + PRP. All the patients had a history of temporomandibular joint pain and/or limited mouth opening. They all signed informed consent forms.

#### 2.1.1 Inclusion criteria

1) clinical symptoms of temporomandibular joint pain, limited mouth opening, and noise upon mouth opening; 2) an X-ray showing condylar bone destruction; 3) aged ≥15 years; and 4) able to undergo postoperative follow-up.

#### 2.1.2 Exclusion criteria

1) suffering from mental illness; 2) a history of trauma in the temporomandibular joint area; 3) oral and maxillofacial or systemic malignant tumors; 4) a history of orthognathic surgery or other oral and maxillofacial surgery; 5) a history of hematological diseases or other systemic diseases; 6) pregnant; 7) with various infectious diseases; and 8) unable to undergo follow-up.

### 2.2 Study method

**FIGURE 1:**
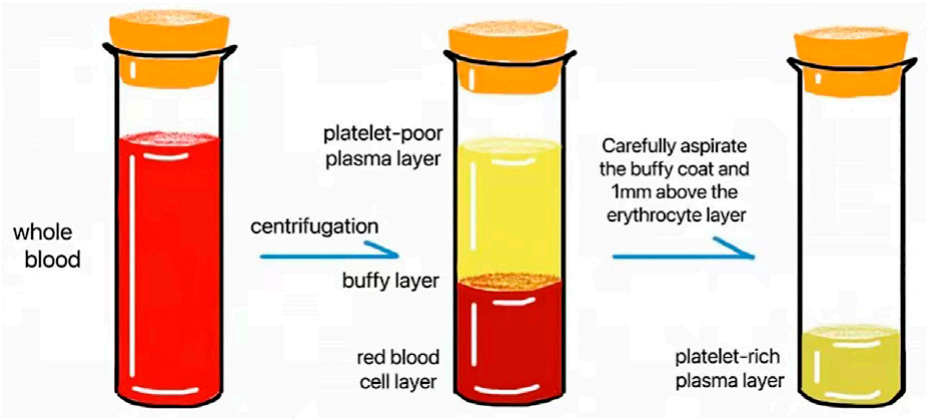
Extraction of PRP.

Preparation before PRP injection:

1. Perform relevant blood tests, including routine blood tests, coagulation factors, hepatitis B and other infectious diseases;
2. Avoid injecting women during their menstrual period;
3. Avoid injecting patients with poor physical conditions, such as colds;

After the contraindications were excluded, 10 ml venous blood was collected in a tube with sodium citrate. An even number of tubes were placed symmetrically in the centrifuge, the PRP mode was selected (1,500 rpm, 10 min), and centrifugation was started. At the end of centrifugation, the blood collection tube was broadly divided into three layers from top to bottom: the platelet-poor plasma layer, the platelet-rich plasma layer and the red blood cell layer. Approximately 1 ml–2 ml of platelet-rich plasma was collected for future use (Figure 1).

### 2.3 Injection method

**FIGURE 2:**
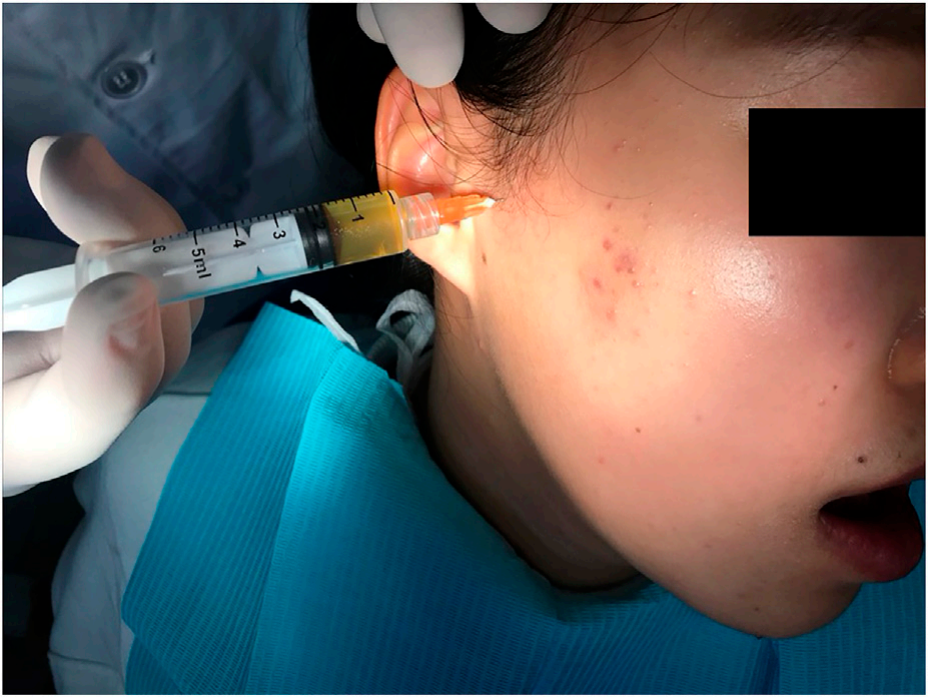
Injection of PRP.

The line between the tragus and lateral canthus, 1 cm in front of the tragus and approximately 0.5 cm vertically downwards were used as the needle insertion mark points (Sha-Sha et al., 2022). Then, 1–2 ml PRP was injected into the articular cavity (Figure 2). The splint was applied to ease the discomfort caused by the 1–2 ml PRP injection. The superficial temporal artery is located in the superior anterior of the external auditory door and can be touched with a finger. The auriculotemporal nerve accompanies it. These areas are avoided during the injection. To ensure the accuracy of the injection site, 0.5 ml PRP was injected first. If it could be totally pulled back then it was certain that the needle was in the upper cavity. No surface anesthesia was applied. We believe that anesthesia, such as lidocaine, may interact with PRP. One time injection of PRP is ok.

**FIGURE 3:**
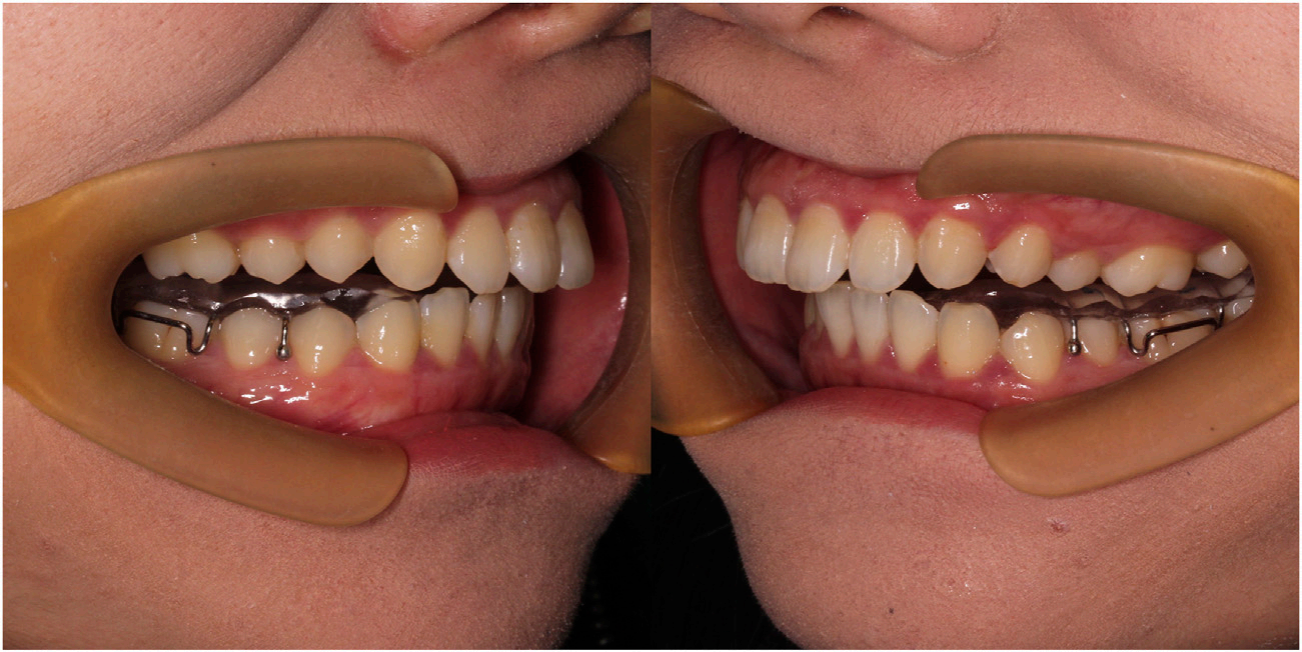
Wearing stability splint.

Splints were uniformly applied by orthodontists in the “joint-orthodontic-orthognathic” group of the Affiliated Stomatological Hospital of China Medical University. The type of splint is a stability splint and 6 months of wearing is required (Figure 3). It does not matter whether it is maxillary or mandibular splint.

The visual analog scale (VAS) was used to evaluate the patient’s average pain, murmur and other problems (Wewers and Lowe, 1990). The patients were informed that 0 means “no pain” and 10 means “the worst excruciating pain.” The VAS scores were self-reported and recorded 6 months after treatment.

The patients were asked to open their mouths as much as possible to ensure comfort. The distance between the incisal end of the maxillary central incisor and the incisal end of the mandibular central incisor was measured with a ruler and recorded.

If the maximum comfortable mouth opening (MCMO) was larger than pretreatment and the posttreatment pain VAS index was lower, treatment was considered to be effective.

A paired *t*-test using SPSS software was performed to analyze all the data, and a *p*-value < 0.05 indicated statistically significant differences.

## 3 Results

**FIGURE 4:**
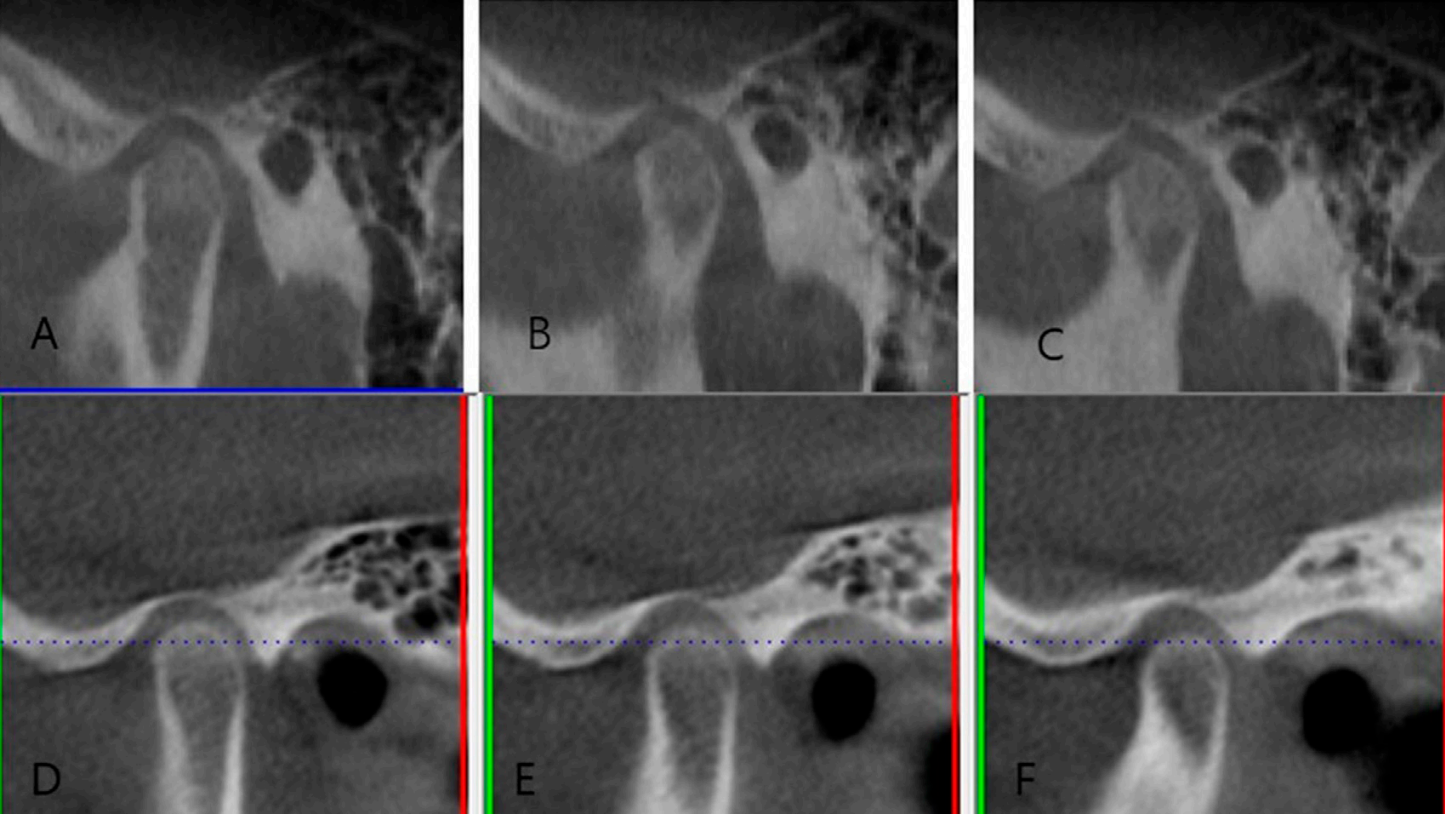
**(A–C)** Imaging manifestations of the left condylar process before treatment. **(D–F)** Imaging manifestations of the left condylar process after Splint + PRP treatment.

Thirty-one patients (thirty-nine joints) were recruited in the cohort, with 24 females and seven males accounting for 77.4% and 22.6%, respectively. The age ranged from 15 to 41 years old, with an average of 26.9 years old. The disease course ranged from 1 to 12 months, with an average of 4.87 months. The MCMO ranged from 18 to 37 mm, with an average of 27.6 mm at pretreatment. The MCMO ranged from 29 to 41 mm, with an average of 34.8 mm at posttreatment. The VAS scores ranged from 5 to 7, with an average of 6.1 at pretreatment. The VAS score ranged from 2 to 8, with an average of 4.1 at posttreatment. After treatment, 27 patients (87.1%) had a larger MCMO, and 27 patients (87.1%) had a lower VAS score (Table 1). The continuity of cortical bone recovered significantly compared with pretreatment ones in some patients (Figure 4).

**TABLE 1:** Patient characteristics.

No.	Age	Gender	Duration (month)	Affected sides	Intervention	Satisfied	Maximum comfortable mouth opening (mm)		Pain	VAS
							Before	After	Before	After
1	23	Female	2	L	Splint + PRP	Yes	22	35	7	4
2	21	Female	3	R	Splint + PRP	Yes	18	31	7	5
3	24	Male	1	R	Splint + PRP	Yes	18	35	7	4
4	27	Female	5	L	Splint + PRP	Yes	23	35	6	3
5	36	Female	8	B	Splint + PRP	Yes	24	32	6	4
6	20	Female	6	R	Splint + PRP	Yes	24	33	6	3
7	15	Female	1	L	Splint + PRP	Yes	35	41	5	2
8	33	Male	2	L	Splint + PRP	Yes	35	39	5	2
9	30	Female	10	B	Splint + PRP	No	35	35	6	8
10	19	Female	2	R	Splint + PRP	Yes	20	35	6	4
11	35	Male	11	R	Splint + PRP	No	37	37	5	7
12	20	Female	3	L	Splint + PRP	Yes	22	32	6	3
13	22	Female	6	B	Splint + PRP	Yes	18	29	6	4
14	24	Female	5	R	Splint + PRP	Yes	21	32	7	4
15	21	Female	7	R	Splint + PRP	Yes	31	33	7	5
16	33	Female	9	B	Splint + PRP	No	34	34	5	7
17	40	Male	5	L	Splint + PRP	Yes	35	37	6	4
18	18	Male	6	L	Splint + PRP	Yes	26	36	6	4
19	30	Female	8	B	Splint + PRP	Yes	31	35	7	4
20	25	Female	2	L	Splint + PRP	Yes	24	36	6	4
21	22	Female	1	R	Splint + PRP	Yes	31	36	7	4
22	41	Female	6	B	Splint + PRP	Yes	26	34	7	4
23	34	Female	5	B	Splint + PRP	Yes	32	36	6	4
24	31	Female	4	R	Splint + PRP	Yes	33	36	5	3
25	25	Female	12	L	Splint + PRP	No	35	35	5	7
26	21	Male	5	R	Splint + PRP	Yes	27	36	6	3
27	25	Female	3	B	Splint + PRP	Yes	35	37	5	3
28	31	Female	2	L	Splint + PRP	Yes	18	30	6	4
29	33	Male	5	R	Splint + PRP	Yes	32	36	5	3
30	19	Female	4	R	Splint + PRP	Yes	29	35	7	4
31	36	Female	2	R	Splint + PRP	Yes	26	36	7	3

The paired *t*-test showed a significant difference between the posttreatment and pretreatment results (*p* < 0.05). The posttreatment VAS scores were significantly lower than the pretreatment VAS scores (*p* < 0.05). The posttreatment MCMO was significantly larger than the pretreatment MCMO (*p* < 0.05).

## 4 Discussion

A splint, similar to retainers or removable dentures, is a device worn on the teeth and is the first and most commonly used device for TMD. Splints are removable in conservative treatment, relieve original occlusal interference, improve muscle function, and ameliorate TMD symptoms after wearing. Splints are widely used in the treatment of temporomandibular joint osteoarthritis because of low cost and removability (Zhang et al., 2016; Robertvan GrootelBuchner et al., 2017; Bergmann et al., 2020). However, the therapeutic mechanism of splinting is still unclear. Most patients with TMJ-OA have poor oral habits (Su et al., 2018), and the author believes that the main purpose of wearing a splint is to counteract the imbalanced stress distribution in the joint area. The stability splint can balance the occlusion and increase the distance between the upper and lower jaws, thereby relieving excitement or fatigue of the masticatory muscles and adjusting the temporomandibular joint to achieve a suitable state, restore normal joint space, and relieve abnormal pressure in the joint area. Thus, the abnormal stress on the joint area is naturally reduced.

At present, there are many treatment methods for TMJ-OA; for example, oral drugs, ultrashort wave physical therapy and other treatment methods, but their long-term efficacy will decrease significantly with time, and long-term use of oral diclofenac sodium and glucosamine may lead to a series of adverse reactions, such as gastrointestinal bleeding (Ouanounou et al., 2017). Therefore, scholars have begun to explore a better alternative therapy, namely temporomandibular joint injection therapy. Although an intra-articular injection is more efficacious and causes fewer adverse reactions than other treatments, there are some differences in the clinical effects of different injected drugs in the treatment of TMJ-OA. For example, studies have shown that glucocorticoids, as the main drug, will accumulate in the joint cavity after long-term and multiple injections, resulting in cartilage and subchondral bone destruction (Habib et al., 2010; Gagé et al., 2016). Therefore, there is a need to find new and safe drugs with few side effects, significant efficacy and stability for TMJ-OA injection therapy.

Platelet-rich plasma is a blood product obtained by centrifuging and separating autologous whole blood (Alsousou et al., 2015). It contains a variety of autologous growth factors and is rich in high concentrations of platelets. It is essentially autologous and has a high concentration of platelets. PRF is a second-generation platelet concentrate product after platelet-rich plasma. Because of its unique fibrin network structure and its richness in platelets and various growth factors, it has a strong role in promoting tissue regeneration. In addition, PRF also has large amounts of immune factors and immune cells that can fight both inflammation and infection and promote tissue healing. PRP has growth factors such as platelet-derived growth factor (PDGF), vascular endothelial growth factor (VEGF),transforming growth factor beta 2 (TGF-β2) and insulin-like growth factor (IGF) (Chandra et al., 2021). Polypeptide growth factors (PGFs) are thought to play an important role in the growth and differentiation of cells involved in periodontal wound healing. They can modulate biological activities in bone and connective tissue, including proliferation, adhesion, migration, and cell differentiation. PRP plays a vital role in cartilage repair, and relevant literature on this role is rich (Marmotti et al., 2015). However, the specific key factors in PRP that regulate and repair chondrocytes are controversial. We do not fully understand the specific regulatory mechanism of PRP. However, we propose that the complicated mechanism commonly regulates the repair process. PRP contains leukocytes, which play an anti-inflammatory role (Scott et al., 2019; David et al., 2009). Compared with PRP, PRF has many characteristics: PRF can delay the release of growth factors and prolong the action time of growth factors because of its viscous effect; and PRF contains a large amount of leukocytes. At the application site, PRF has anti-inflammatory and anti-infection capabilities. PRF has a loose fibrin structure, and there are gaps between molecules, which is conducive to cell crawling, growth, proliferation and differentiation. PRF has a certain stability due to its shape and can induce bone formation. It can be used alone to induce osteogenesis or combined with bone substitute materials to enhance support (Pietruszka et al., 2021).

In several hypotheses about platelets, the mechanism of antimicrobial action is better understood. Platelets play a key role in the immune system. They secrete molecules that have a defensive effect on the body. After stimulation with thrombin, platelets release specialized proteins with inhibitory bacterial and fungal activity, such as PDGF, TGF-β, and IGF. These factors support biological processes necessary for normal healing and regeneration. They also generate reactive oxygen species (ROS), bind microorganisms and participate in antibody-dependent cellular cytotoxicity (ADCC). Platelets are involved in the identification and neutralization of pathogenic microorganisms. They also recruit leukocytes to sites of infection and inflammation and regulate their function (Shubhra Bhattacharya et al., 2020; Pietruszka et al., 2021; Manafikhi et al., 2022).

In the clinical setting, PRP is commonly combined with other methods. Researchers have compared the efficacy of arthrocentesis combined with hyaluronic acid injection with arthrocentesis combined with PRP injection. The authors found no significant difference in clinical outcomes between the two groups of patients (Comert and Gungormus, 2016; Yuce and Komerik, 2020). Arthrocentesis with multiple injections of PRP is not superior to arthrocentesis with an injection of hyaluronic acid. In this study, we proposed a new treatment strategy: splint combined with PRP. The results showed that treatment with splint + PRP significantly increased MCMO and decreased the VAS score for pain. In this study, we used the MCMO and pain VAS index to evaluate efficacy without imaging data. Morphological and histological examinations are still expensive for many patients. In addition, the following considerations should be kept in mind. Efficacy was determined by the success of the treatment. The patient comes to the clinic in urgent need of pain relief and improved mouth opening. If pain is relieved and mouth opening is improved, then these outcomes are sufficient to evaluate treatment efficacy. Therefore, from our perspective, the success or failure of TMJ-OA treatment depends on the main complaint of the patient. If the VAS score was lower and the mouth opening capacity was greater, then the treatment was considered to be successful. We collected cases according to the established inclusion and exclusion criteria. After treatment, some patients were followed up, and some patients were lost to follow-up. To ensure the integrity of case collection, we established postoperative follow-up as one of the inclusion criteria. If the patient could not be followed up, we did not obtain postoperative metrics. To ensure the accuracy of the research, we excluded interference factors. Patients with systemic diseases and pregnant women were excluded if there was a sufficient number of healthy cases. Frankly speaking, thirty-nine patients came to our hospital from January 2021 through June 2021. However, given the inclusion and exclusion criteria, thirty-one patients were recruited finally. Maximum mouth opening was improved in more than 80% of patients in the splint + PRP group. The above results show the therapeutic effect of splint + PRP.

Injectable PRP or splint is an important treatment modality for TMD. As we mentioned previously, our team is focusing on the research of Piezo1. We found that unbalanced stress may activate Piezo1, thus leading to TMJ-OA. On the other hand, the inflammatory environment may activate Piezo1, aggravating TMJ-OA. Given such conditions, removing abnormal stress loading and eliminating inflammation are both vital for the treatment of TMJ-OA. So, we take it for granted that the splint combined with injectable PRP is better than single PRP or single splint. The study has shortcoming because of no control group. But, we used paired *t*-test to assess the therapeutic effect. As we know, the paired *t*-test, a self-controlled statistical method that can eliminate interference associated with outcome variables, was applied. This study focus its attention on the treatment effect of PRP combined with splint. To the best of our knowledge, this is the first study in which researchers assess the effectiveness of splint + PRP treatment. However, there are some limitations of the study. First, the sample size was small. Only 31 patients were studied. Second, the follow-up time was short. Although a therapeutic effect may be apparent in 6 months, a long-term follow-up would yield more thorough results. Third, the evaluation indicators were not efficient. We recorded pain only at baseline and 6 months after treatment. The pain after injection and in between was not recorded.

We developed a new and effective conservative treatment. We look forward to verifying its effectiveness in various countries and groups in future studies. Although the number of patients who recovered in the splint + PRP group was greater than that in the other two groups, there were still some patients whose symptoms were not alleviated. This lack of improvement confirms that the treatment also has its shortcomings. New conservative treatments should be explored in the future, such as a combination with physical therapy.

## Data Availability

The original contributions presented in the study are included in the article/supplementary material, further inquiries can be directed to the corresponding author.
